# Dynamics of Intestinal Mucosa Microbiota in Juvenile Sika Deer During Early Growth

**DOI:** 10.3390/ijms26030892

**Published:** 2025-01-22

**Authors:** Songze Li, Ruina Mu, Yunxi Zhang, Shaoying Wang, André-Denis G. Wright, Huazhe Si, Zhipeng Li

**Affiliations:** 1 College of Animal Science and Technology, Jilin Agricultural University, Changchun 130118, China; 2 School of Biological Sciences, University of Oklahoma, Norman, OK 73019, USA; 3 Joint International Research Laboratory of Modern Agricultural Technology, Ministry of Education, Jilin Agricultural University, Changchun 130118, China; 4 Key Laboratory of Animal Production, Product Quality and Security, Ministry of Education, Jilin Agricultural University, Changchun 130118, China; 5 Jilin Provincial Engineering Research Center for Efficient Breeding and Product Development of Sika Deer, Jilin Agricultural University, Changchun 130118, China

**Keywords:** mucosal microbiota, small intestine and large intestine, juvenile sika deer

## Abstract

The establishment of gut microbiota in young ruminants has a profound impact on their productive performance in adulthood. The microbial communities of ruminants differ significantly across the different regions of the digestive tract, as well as between the mucosa and lumen. In this study, we analyzed the characteristics of the microbiota of the small intestine (jejunum and ileum) and large intestine (cecum and colon) of sika deer on day 1 (birth), day 42 (transition period) and day 70 (rumination period) using 16S rRNA gene sequencing. The results showed that the microbial diversity of the mucosa in the jejunum, ileum, cecum and colon of sika deer was higher on day 70 than on day 1, and the diversity of the cecal mucosa was significantly higher than that in the small intestine. Principal coordinates analysis (PCoA) showed that the microbial community structures of the small and large intestinal mucosa were significantly different, and the microbial community structure of sika deer on day 1 was significantly different from that on days 42 and 70. The relative abundances of *Methylobacterium–Methylorubrum*, *Pelagibacterium*, *Acinetobacter* and *Staphylococcus* were higher in the small intestinal mucosa, while *Alistipes*, Prevotellaceae UCG-004, *Eubacterium coprostanoligenes* group and Lachnospiraceae unclassified were higher in the large intestinal mucosa. We also observed increased levels of specific microbiota in the small intestinal (*Turicibacter* and *Cellulosilyticum*) and large intestinal mucosa (*Treponema*, *Romboutsia*, Oscillospirales UCG-005 and Peptostreptococcaceae unclassified) with animal growth. A comparison of the predicted function showed that the microbiota of the small intestinal mucosa was enriched for replication and repair, while carbohydrate metabolism was enriched in the microbiota of the large intestinal mucosa. In addition, the relative abundances of amino acid and energy metabolism were significantly higher on days 42 and 70 than on day 1. Our results revealed that the microbial community composition and the dynamics of the intestinal mucosa from birth to rumination in juvenile sika deer, which may provide insights into similar processes in other juvenile ruminants.

## 1. Introduction

The gastrointestinal tract (GIT) microbiota of ruminants has developed a close collaboration and symbiotic relationship with the host, particularly in improving host productivity [1], feed efficiency [2] and reducing methane emissions [3]. Recent findings have demonstrated that the GIT microbiota of ruminants become increasingly complex and diverse after birth, and the colonization and succession of these microorganisms significantly impact the health and performance of the host in adulthood [4,5,6]. Malmuthuge et al. (2019) found that gut microbial modulation in newborn calves, especially by *Lactobacillus*, impacts immune function. *Lactobacillus*-dominant calves showed an increased expression of proinflammatory chemokines, suggesting immune priming that prepares the immune system for future anti-inflammatory responses, which is vital for neonatal calves [7]. Fan et al. (2021) found that butyrate-producing bacteria in the hindgut of calves are involved in the regulation of host immune function [8]. Li et al. (2018) found that *Escherichia*–*Shigella* are enriched in the small intestine of sika deer during the neonatal period [9]. These microorganisms are major pathogens that cause diarrhea in young ruminants and are also among the primary causes of intestinal diseases in humans [10]. Therefore, an exploration of the GIT microbial colonization and succession in young ruminants is crucial for optimizing their productive performance.

In China, sika deer is one of the most economically valuable members of the Cervidae family because it is used for the production of velvet antler, a precious Chinese medicine [11]. It was revealed that the GIT microbiota of adult sika deer exhibited segmental characteristics, with *Prevotella* representing the core microbiota of the stomach and Christensenellaceae R-7 being predominantly present in the intestine, and the microbial diversity of the large intestine was significantly higher than that of the stomach [12]. However, 60 days after weaning, the rumen of juvenile sika deer is still underdeveloped, the intestinal microbiota plays important roles in digesting and absorbing the ingested nutrients [13]. The small intestinal microbiota mainly absorb proteins and carbohydrates [14], whereas plant cellulose and oligosaccharides are usually fermented by the large intestinal microbiota to produce short-chain fatty acids (SCFAs) as an energy source for the host [15]. Our previous studies found that the birth–transition period is a critical phase for the establishment of the gut bacterial community and metabolic function of juvenile sika deer [6]. Importantly, we observed increases in the proportion of Ruminococcaceae UCG-005, Ruminococcaceae UCG-010, Rikenellaceae RC-9 and Prevotellaceae UCG-003 and the concentration of total SCFAs in the cecal and colonic lumen with juvenile sika deer growth [16]. Moreover, the ruminal passage rate of feed in sika deer was comparatively higher than that in cattle [17]. These results further suggest the important roles of the cecal and colonic microbiota of juvenile sika deer.

However, previous studies demonstrated that the number of operational taxonomic units (OTUs) in mucosal tissues of the jejunum, ileum, cecum and colon were higher than the respective luminal content in adult dairy cows [18]. The intestinal lumen microbiota plays a role in the digestion and metabolism of nutrients, and its composition is more heterogeneous, which is influenced by the distribution of undigested food and plant and starch particles, which provide attachment sites for bacteria [19]. Intestinal mucosal microbiota plays a significant role in the regulation of the immune system and the maintenance of epithelial barrier integrity [19]. Similarly, the composition of the microbial communities of the intestinal lumen and mucosa was found to be significantly different from that of pre-weaning calves and lambs [20,21,22,23]. Theabundance of Lactobacillaceae was higher in the cecal and colonic mucosa than in the luminal contents of the intestine, but was lower in the jejunal and ileal mucosa. Compared to luminal microbiota in the intestine, microbial diversity and richness in the mucosa were higher, and microbial load was lower in goat kids and dairy calves [24,25]. However, the changes in the microbial community in the intestinal mucosa of juvenile sika deer remain unclear.

In this study, we analyzed the changes in intestinal mucosa microbiota and predicted function using 16S rRNA sequencing of the sika deer on day 1 (birth), day 42 (transition period) and day 70 (rumination period), laying the basis for further understanding of the development of intestinal mucosal microbiota in juvenile sika deer.

## 2. Results

### 2.1. Taxonomic Composition of Intestinal Mucosal Microbiota

**Figure 1:**
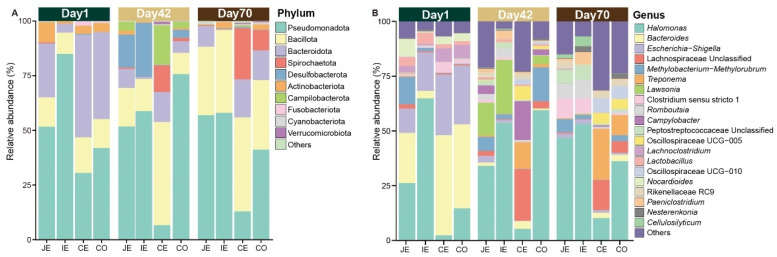
Microbial community composition in jejunal (JE), ileal (IE), cecal (CE) and colonic (CO) mucous of juvenile sika deer on day 1, day 42 and day 70 at the phylum (**A**) and genus (**B**) levels.

A total of 1,582,020 high-quality reads were obtained from the jejunal, ileal, cecal and colonic mucous on day 1, day 42 and day 70, with an average of 26,367 reads per sample, which were further clustered into 2511 amplicon sequence variants (ASVs). Taxonomic classification identified a total of 16 phyla that were dominated by the phyla Pseudomonadota, Bacillota, Bacteroidota, Spirochaetota and Desulfobacterota in all groups (Figure 1A). The 2511 ASVs were further classified into 297 genera. In the jejunal mucosa, the genera *Halomonas*, *Bacteroides*, *Methylobacterium*–*Methylorubrum* and *Escherichia*–*Shigella* were the most prevalent genera. In the ileal mucosa, the genera *Halomonas*, *Escherichia*–*Shigella*, *Lawsonia* and *Romboutsia* were the most prevalent genera. In the cecal mucosa, the genera *Bacteroides*, Lachnospiraceae unclassified, *Treponema* and *Escherichia–Shigella* were the most prevalent genera. In the colonic mucosa, the genera *Halomonas*, *Bacteroides*, *Escherichia–Shigella* and *Methylobacterium–Methylorubrum* were the most prevalent genera (Figure 1B and Table S2).

### 2.2. Changes in Microbial Diversity and Communities in Intestinal Mucosa

**Figure 2:**
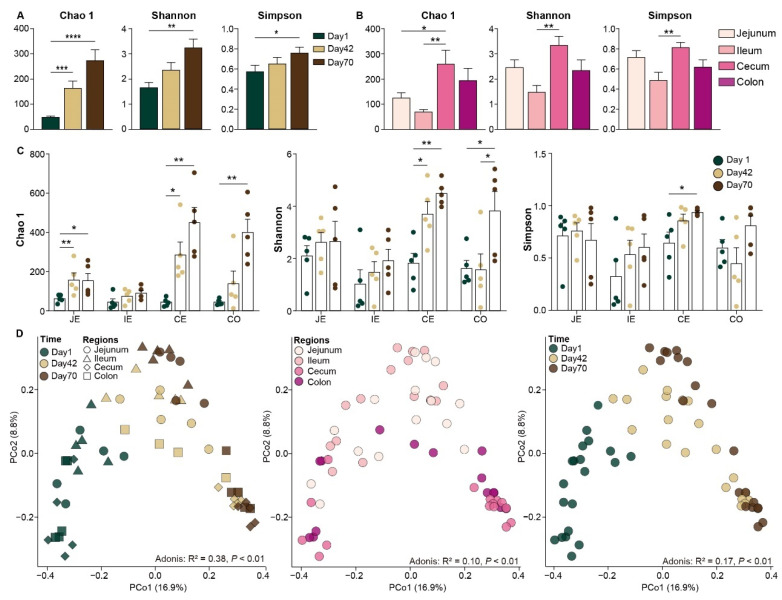
Comparison of microbial community in mucosa among the different intestinal regions and time points. Comparison of microbial diversity indices of juvenile sika deer among the three time points (**A**), and among the different intestinal regions (**B**). Comparison of diversity indices on day 1, day 42 and day 70 for mucosa in each intestinal region (**C**). PCoA revealing the microbial community and composition of intestinal mucosa based on unweighted Unifrac distance (**D**). *, **, ***, and **** indicate *p* < 0.05, *p* < 0.01, *p* < 0.001 and *p* < 0.0001, respectively. JE, jejunum; IE, ileum; CE, cecum; CO, colon.

We compared the microbial diversity and found a higher microbial diversity on day 70 than on day 42 or day 1, as indicated by the significant differences of the Chao1, Shannon and Simpson indices (*p* < 0.05, Figure 2A). In addition, the microbial diversity of the cecal mucosa was significantly higher than that of the ileal mucosa (*p* < 0.05, Figure 2B). The diversity indices (Chao1 and Shannon indices) of the cecal and colonic mucosa were significantly higher on day 70 than on day 1 (*p* < 0.05, Figure 2C). The PCoA showed that the microbial communities of the small intestinal (jejunum and ileum) mucosa and large intestinal (cecum and colon) mucosa were clearly separated based on the Bray–Curtis dissimilarity matrix and weighted and unweighted Unifrac distance; the microbial community on day 1 was clearly different from those on day 42 and day 70 (*p* < 0.01; Figure 2D and Figure S1A,B). Furthermore, the difference between the mucosal microbiota of the small and large intestines was much clearer on day 70 in comparison with day 1 and day 42 (Figure S1C), whereas the separation of the mucosal microbiota on day 1, day 42 and day 70 was more significant in the large intestine than in the small intestine (*p* < 0.01, Figure S1D and Table S3).

### 2.3. Changes in Microbial Composition of Intestinal Mucosa on Day 1, Day 42 and Day 70

**Figure 3:**
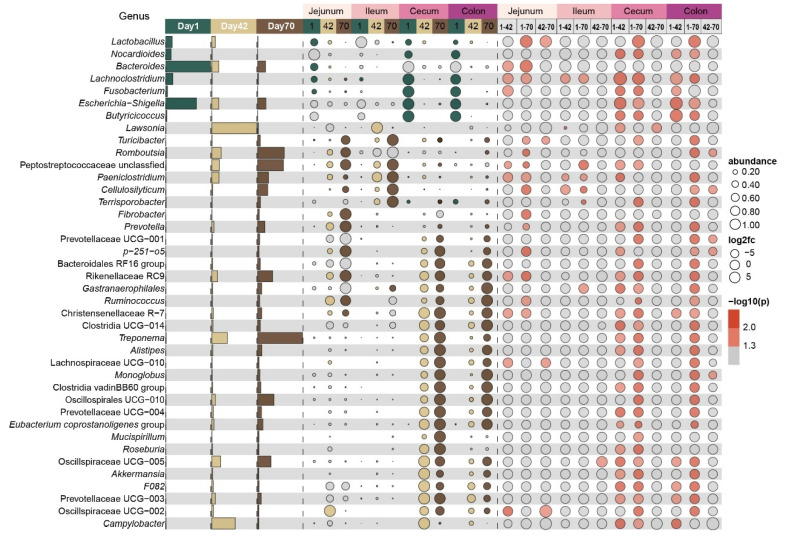
The significantly different genera in mucosa of each intestinal region at three time points. Bar graphs showing the average relative abundances of microbiota at the genus level on day 1 (green), day 42 (yellow) and day 70 (brown). Bubble graphs showing significantly changed genera, significance and fold change for three age groups in the jejunum (light pink), ileum (pink), cecum (dark pink) and colon (purple), respectively. The numbers 1, 42 and 70 represent day 1, day 42 and day 70, respectively.

We further found that a total of 17, 8, 36 and 34 genera were significantly different in the jejunal, ileal, cecal and colonic mucosa, respectively, among day 1, day 42 and day 70 (*p* < 0.05, Figure 3 and Tables S4–S7). In the jejunal and ileal mucosa, the relative abundances of *Turicibacter* and *Cellulosilyticum* were significantly higher on day 70 than on day 1. Moreover, the relative abundances of *Lactobacillus*, *Lachnoclostridium* and *Bacteroides* in the jejunal mucosa were significantly higher on day 1 than on day 42 or day 70. In the cecal mucosa, the relative abundances of *Treponema*, Oscillospirales UCG-005 and Peptostreptococcaceae unclassified were significantly higher on day 70 than on day 1. In the colonic mucosa, the relative abundances of *Treponema*, *Romboutsia* and Oscillospirales UCG-005 were significantly higher on day 70 than on day 1. In the ileal mucosa, the relative abundances of *Lawsonia* were significantly higher on day 42 than on day 1.

### 2.4. Changes in Microbial Composition in Different Intestinal Regions at the Same Time Points

**Figure 4:**
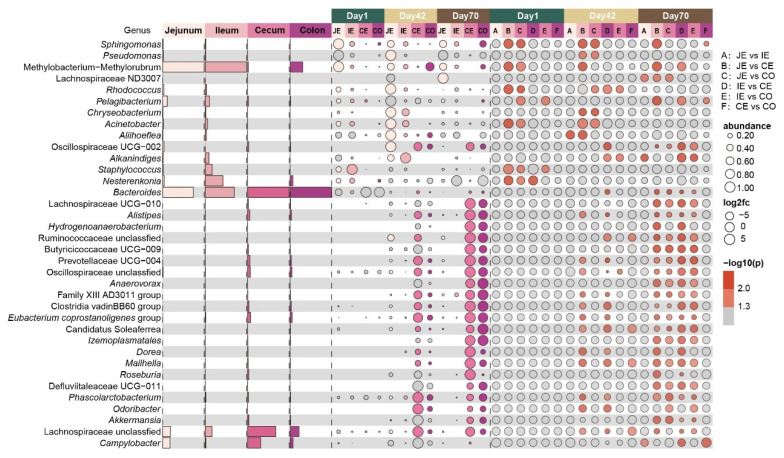
Differences in intestinal mucosal microbiota in different intestinal regions at the same time points. Bar graphs showing the average relative abundances of genera in the jejunum (light pink, JE), ileum (pink, IE), cecum (dark pink, CE) and colon (purple, CO). Bubble graphs showing the significantly changed genera, significance and fold change for different intestinal regions on day 1 (green), day 42 (yellow) and day 70 (brown), respectively.

We also examined the difference in mucosal microbiota in the different intestinal regions on day 1, day 42 and day 70 (*p* < 0.05, Figure 4 and Tables S8–S10). A total of 7, 22 and 29 genera were found at each time point, respectively. On day 1, the relative abundances of *Methylobacterium–Methylorubrum*, *Pelagibacterium*, *Acinetobacter* and *Staphylococcus* were significantly higher in the jejunal mucosa than in the cecal mucosa or colonic mucosa. In addition, the relative abundances of *Pelagibacterium* and *Staphylococcus* were significantly higher in the ileal mucosa than in the colonic mucosa. On day 42, the relative abundances of *Bacteroides*, *Alistipes*, Prevotellaceae UCG-004, *Eubacterium coprostanoligenes* group and Lachnospiraceae unclassified were significantly higher in the cecal mucosa than in the jejunal mucosa or ileal mucosa. On day 70, the relative abundances of *Pelagibacterium* and *Methylobacterium–Methylorubrum* were greater in the ileal mucosa than in the cecal mucosa, while bacteria including *Bacteroides*, *Alistipes*, Prevotellaceae UCG-004, *Eubacterium coprostanoligenes* group and Lachnospiraceae unclassified were enriched in the cecal mucosa or colonic mucosa.

### 2.5. Changes in the Predicted Functional Profiles of the Intestinal Mucosal Microbiota

**Figure 5:**
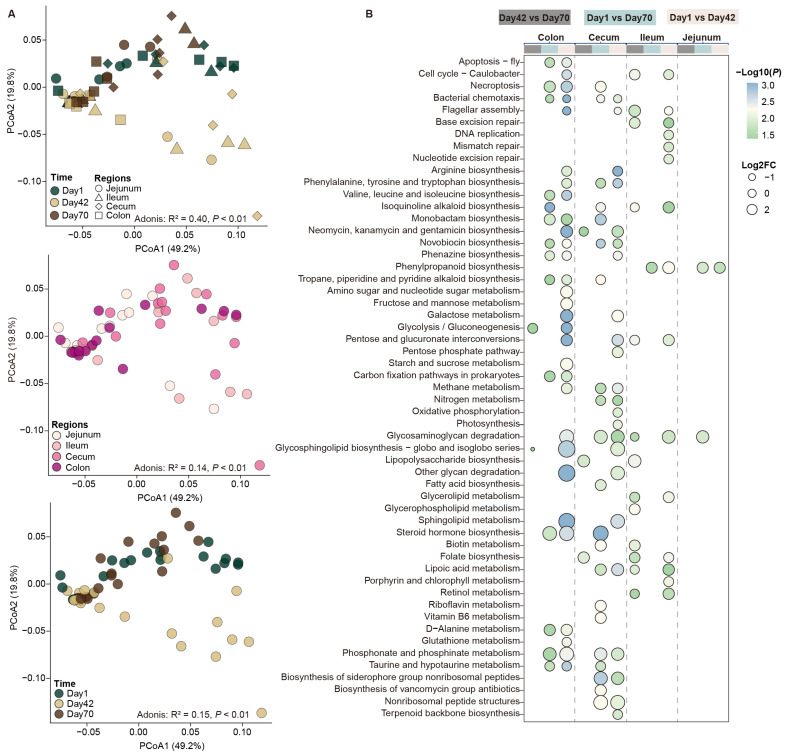
Comparisons of intestinal mucosal microbial function profiles. PCoA results revealing the variation of the difference of the predicted functions of mucosal microbiota in the different intestinal regions and at the three stages at KEGG level 3 based on the Bray–Curtis dissimilarity matrix (**A**). The significantly different pathways at the three stages at KEGG level 3 (**B**). The circle size represents the log2 fold change (FC) values of day 42 vs. day 70, day 1 vs. day 70 and day 1 vs. day 42. The circle color indicates the logarithm of *p* values calculated as the fraction of permutation values. The color legend at the left of the heat map indicates the different comparisons. The significances were determined via a Benjamini–Hochberg-adjusted *p* value < 0.05 using relative abundances of KEGG level 3.

We then predicted the potential functions of the intestinal mucosal microbiota and found significant divergence of the metabolic pathways in different intestinal regions and on day 1, day 42 and day 70 (Bray–Curtis dissimilarity matrix, *p* < 0.01, Figure 5A). A total of 55 pathways at KEGG level 3 were significantly different in the intestinal mucosa at the three time points (*p* < 0.05, Figure 5B). For instance, the relative abundances of carbohydrate metabolism (galactose metabolism, starch and sucrose metabolism, and glycolysis/gluconeogenesis) and lipid metabolism (fatty acid biosynthesis and glycerolipid metabolism) were significantly increased on day 42 and day 70 compared with those on day 1. Further comparisons of the mucosal microbial function in each intestinal region showed that the jejunal mucosal microbiota were mainly enriched in biotin metabolism, retinol metabolism and fatty acid biosynthesis. The relative abundances of base excision repair, DNA replication and mismatch repair were significantly higher in ileal mucosal microbiota. In addition, valine, leucine and isoleucine biosynthesis, amino sugar and nucleotide sugar metabolism, and galactose metabolism were significantly increased in the cecal and colonic mucosa.

## 3. Discussion

Here, we revealed the dynamics of microbial communities of the jejunal, ileal, cecal and colonic mucosa of juvenile sika deer from birth (day 1) to the rumination periods (day 70). The results showed that Pseudomonadota, Bacteroidota and Bacillota were the predominant phyla in the intestinal mucosa of juvenile sika deer, which is consistent with observations in lamb [23], goat kids [26] and dairy cows [27]. Members that belong to the Pseudomonadota phylum are facultative anaerobes that colonize the gut of dairy cows at birth and are gradually replaced by members of the Bacteroidota and Bacillota phyla [28]. Previous observations have shown that a higher abundance of Pseudomonadota in the gut can reflect the instability of the microbial community structure and is also a key indicator of intestinal health in mammals [29]. These results suggest that a high abundance of Pseudomonadota favors the colonization of anaerobic bacteria in the intestinal mucosa. Our study also found increased relative abundances of the phyla Bacillota and Spirochaetota in the intestinal mucosa from day 1 to day 70. Members of the phylum Bacillota, such as Ruminococcaceae, Christensenellaceae and Lachnospiraceae, were found to provide energy for the host by degrading cellulose [30,31]. It has been demonstrated that members belonging to the phylum Spirochaetota could promote protein and cellulose degradation [32]. These findings suggest that the ability to degrade nutrients in the intestinal mucosa increases with juvenile sika deer growth.

Our results also showed the significant effects of time points and intestinal regions on microbial diversity. The diversity of mucosal microbes on day 70 was higher than that on day 42 and day 1, agreeing with previous findings that the GIT mucosal microbial diversity increases significantly with goats’ growth [33]. Previously, we also found that the microbial diversity of the small and large intestinal contents increased with juvenile sika deer growth [34]. These results demonstrate the common prosperity of intestinal content and mucosal microbial community during the early growth of juvenile sika deer. In addition, we found that the microbial diversity of the cecal mucosa was higher than that of the small intestinal mucosa. Similar results were found in pre-weaned calves [20]. The microbial diversity of the cecum and colon was found to be greater than that of the small intestinal content of adult sika deer [12]. The small intestine is the core site for the absorption of nutrients, its physiological environment is different from that of the large intestine because of the low pH and shorter transit times, and is unfavorable for microbial survival [12,35]. Importantly, the high ruminal passage rate of feed in sika deer, combined with the greater proportion of hindgut length to total intestinal length, prolongs feed retention time in the hindgut, allowing for further fermentation of unfermented material from the rumen [17]. The composition of the microbial communities in the digestive tract of water deer and roe deer showed significant differences between regions and under different dietary conditions [36]. These results suggest the important role of the physiological environment in determining mucosal microbiota.

Our results showed that the dominant genera in the intestinal mucosa on day 1 included *Lactobacillus*, *Bacteroides* and *Lachnoclostridium*. Previous studies demonstrated that intestinal microbiota can originate from the mother’s vagina [37,38] and the breast milk [22,39,40]. *Bacteroides* can degrade breast milk oligosaccharides via mucosa utilization pathways [41], and *Lactobacillus* can hydrolyze lactose from breast milk to lactate via lactase [42]. While *Lachnoclostridium* can further metabolize lactate to produce SCFAs [43], which provide energy for intestinal epithelial cells to grow. Recent findings demonstrated that complement components from breast milk shape neonate and infant gut microbial composition by selectively eliminating members of the commensal gut community [44]. These results suggest that the establishment of microbiota in the intestinal mucosa of juvenile sika deer are likely affected by breast milk components. In addition, we found that the abundances of *Turicibacter* and *Cellulosilyticum* in the jejunal and ileal mucosa were significantly higher on day 70 than on day 1. *Turicibacter* plays a crucial role in regulating gastrointestinal immunity [45] and is actively involved in bile acid metabolism [46]. *Cellulosilyticum* exhibits a robust cellulose-degrading capacity and secretes cellulases that hydrolyze plant cellulose into simpler sugars, facilitating the breakdown of complex polysaccharides in plant cell walls [47]. Moreover, the relative abundances of genera (*Treponema*, *Romboutsia*, Oscillospirales UCG-005 and Peptostreptococcaceae unclassified) related to the utilization of carbohydrates for SCFA production were higher in the cecal mucosa and colonic mucosa on day 70 [18,48,49]. Interestingly, we previously found that the immune responses of the epithelium and mucosa of the small intestine are enhanced, but by different mechanisms regulated by SCFAs [34]. These results indicate that the role of mucosal microbiota in affecting immune function likely differs in the small and large intestine of juvenile sika deer.

The relative abundances of genera including *Methylobacterium–Methylorubrum*, *Pelagibacterium*, *Acinetobacter* and *Staphylococcus* in the small intestinal mucosa were significantly higher than those in the large intestinal mucosa, and *Escherichia–Shigella* was the most prevalent genus in the small intestinal mucosa. Members of *Escherichia*–*Shigella* are predominant pathogens in the small intestine of neonatal sika deer and are commonly associated with the onset of diarrhea [9]. *Methylobacterium–Methylorubrum* and *Acinetobacter* are regarded as opportunistic pathogens and can induce intestinal inflammation [18,50]. However, they have also been found to promote small intestinal crypt epithelial renewal and positively correlate with peripheral immune function [51,52]. Previous studies have found that *Pelagibacterium* and *Staphylococcus* contribute to optimizing early gut microbial colonization and enhance intestinal resistance to disease and nutrient absorption [53,54]. Amin et al. (2021) observed an early enrichment of microbiota associated with immune regulation in the small intestine of calves [55]. These results suggest the possible key roles of these microbiota of the small intestinal mucosa in competing with pathogenic bacteria for ecological niches, as well as maintaining the integrity of the intestinal epithelial barrier.

In addition, we also found that the relative abundances of *Alistipes*, Prevotellaceae UCG-004, *Eubacterium coprostanoligenes* group and Lachnospiraceae unclassified were significantly higher in the large intestinal mucosa of juvenile deer. Palomba et al. (2017) also observed a higher abundance of Lachnospiraceae in the large intestinal mucosa compared to the small intestine of pre-weaning lambs [23]. Similarly, a significant enrichment of Lachnospiraceae unclassified was also observed in the hindgut of lambs [33]. Prevotellaceae and Lachnospiraceae have been found to promote the digestion of hemicellulose, pectin and high-carbohydrate diets in the hindgut [56,57]. *Eubacterium* plays a crucial role in enhancing nutrient availability [58]. This is consistent with the physiological function of the large intestine, where the microbe mainly ferments plant cellulose and oligosaccharides transferred from the small intestine without digesting or absorbing them [15]. Furthermore, it is known that these dominant genera have the ability to produce butyrate [59,60,61], which is essential for maintaining host intestinal health and providing energy for the intestinal mucosa. These results suggest that the microbiota of the large intestinal mucosa also have an important role in the proliferation and differentiation of intestinal epithelial cells.

In this study, we found that the relative abundances of carbohydrate metabolism and lipid metabolism in the intestinal mucosa were higher at birth, while the relative abundances of amino acid metabolism and energy metabolism were higher during the transition and rumination periods. This is consistent with the findings of Song et al. (2021, 2022) and Kim et al. (2021), who examined the gut microbiota of calves [62,63,64] and suggested that the gut mucosal microbes likely obtain energy from carbohydrates after birth. In addition, our findings also showed that base excision repair, DNA replication, mismatch repair and the cell cycle were significantly enriched in the small intestine, whereas valine, leucine and isoleucine biosynthesis, fatty acid biosynthesis and galactose metabolism were significantly enriched in the large intestine. It has been demonstrated that the enhanced amino acid metabolism characteristics in intestinal microbiota of pre-weaning calves contribute to better nutrient absorption [25]. The relative abundances of carbohydrate metabolism and membrane transport were higher in the cecal and colonic mucosa of goat kids than in the rumen [33]. Our previous findings of the intestinal mucosa of juvenile sika deer [34] showed that the T-cell receptor signaling pathway and Th17 cell differentiation were enriched in the small intestine, and arginine synthesis and metabolism was enriched in the large intestine. This study further demonstrates the role of arginine metabolism in the regulation of intestinal immune responses of juvenile sika deer [34].

## 4. Materials and Methods

### 4.1. Animals and Sample Collection

A total of 15 healthy juvenile sika deer of similar weight from our previous study were used [6], consisting of 5 juvenile sika deer on day 1 (birth), 5 juvenile sika deer on day 42 (transition period) and 5 juvenile sika deer on day 70 (rumination period). The juvenile deer were kept with their dams and weaned on day 60. Before weaning, the juvenile deer were allowed to feed freely, with the ratio of concentrate to corn silage being 50:50 (dry matter basis, Table S1). After weaning, the juvenile deer were fed separately from their dams. Throughout the experiment, all animals had free access to water. All animal-specific procedures were approved and authorized by the Animal Ethics Committee of Jilin Agricultural University (Approval ID: 20220317001).

Each of the five juvenile sika deer were selected for slaughter after morning feeding (3 h) on day 1, day 42 and day 70. The junctions of the jejunum, ileum, cecum and colon were ligated with sterile cotton thread. Epithelial samples of jejunum, ileum, cecum and colon (approximately 8 cm^2^) were collected and washed three times with ice-cold phosphate-buffered saline (pH 7.0). Intestinal mucosa samples were scraped with a sterile slide and immediately snap-frozen in liquid nitrogen, and then were stored at −80 °C until further processing for microbial DNA extraction.

### 4.2. DNA Extraction, 16S rRNA Gene Amplification, Sequencing and Bioinformatic Analysis

The microbial genomic DNA was extracted from each mucosa sample of jejunum, ileum, cecum and colon using the QIAamp^®^ Fast DNA Stool Mini Kit (QIAGEN, Valencia, CA, USA). The primers 341F (5′-CCTACGGGAGGCAGCAG-3′) and 806R (5′-GGACTACHVGGGTWTCTAAT-3′) were used to amplify the V3 to V4 region of the microbial 16S rRNA gene [65]. The PCR products were purified with the QIAquick PCR Purification Kit (QIAGEN, CA, USA). The purified amplicons were then used to construct sequencing libraries. Briefly, the amplicon libraries were prepared by adding appropriate adapters and barcodes for Illumina sequencing. A PhiX control library (Illumina, San Diego, CA, USA, 20%) was combined with the amplicon library to generate paired 250 bp reads and then sequenced using the Illumina PE MiSeq platform.

The FASTP [66] was used to perform an initial filtering of the raw sequences with the following parameters: −q 15 −l 15; then, the trimmed sequences were imported into QIIME 2 [67]. After filtering low-quality sequences and removing chimeras using the chimera.vsearch function in DADA2, which detects and removes chimera sequences based on a reference database, the DADA2 pipeline (https://benjjneb.github.io/dada2/tutorial.html (accessed on 1 June 2024)) was used to identify amplicon sequence variants (ASVs). The ASVs are defined at 100% sequence identity. BLAST was used for sequence alignment, and taxonomic annotation was based on the SILVA database (v138) [68] using a confidence threshold of 0.8. The alpha diversity, including Chao1, Shannon and Simpson indices, was calculated using microeco package (v1.9.0) [69]. The functional profiles of the intestinal mucosal microbial communities were predicted using the Tax4Fun2 [70], which assigns taxonomy to the ASVs and maps them to functional pathways based on the KEGG database to predict potential metabolic functions of the microbial communities. The differences in microbial communities and functions in the intestinal mucosa were revealed by principal coordinate analysis (PCoA) based on Bray–Curtis dissimilarity matrix, weighted UniFrac distance and unweighted UniFrac distance. The group similarity was determined using permutational multivariate analysis of variance (Adonis).

### 4.3. Statistical Analysis

The Kruskal–Wallis (KW) test was used to identify the differences in alpha diversity indices, composition and functions of intestinal mucosal microbiota. The *p* values were adjusted with the false discovery rate of the Benjamini–Hochberg method, and the corrected *p* values ≤ 0.05 were regarded as significantly different.

## 5. Conclusions

Here, we characterized the intestinal mucosal microbiota of juvenile sika deer from birth to the rumination period. The results showed that the microbial diversity indices of the intestinal mucosa were significantly increased with age, and the mucosal microbial diversity of the small intestine was lower than that of the large intestine. Different intestinal regions were characterized by distinct microbial compositions. The amino acid metabolism of the microbiota of the large intestinal mucosa represents a pivotal factor in the developmental processes of juvenile sika deer.

## Data Availability

Raw sequence reads for all samples are available under NCBI project PRJNA 1095682.

## Supplementary Materials

The following supporting information can be downloaded at: https://www.mdpi.com/article/10.3390/ijms26030892/s1.
