# Supplementary material for: Dynamics of Intestinal Mucosa Microbiota in Juvenile Sika Deer During Early Growth
Source: Int J Mol Sci. 2025 Jan 22;26(3):892. doi: 10.3390/ijms26030892 (PMC11817005; doi:10.3390/ijms26030892)
Supplement: Supplementary file 1 [file ijms-26-00892-s001.zip › Supplementary Material 1.pdf]

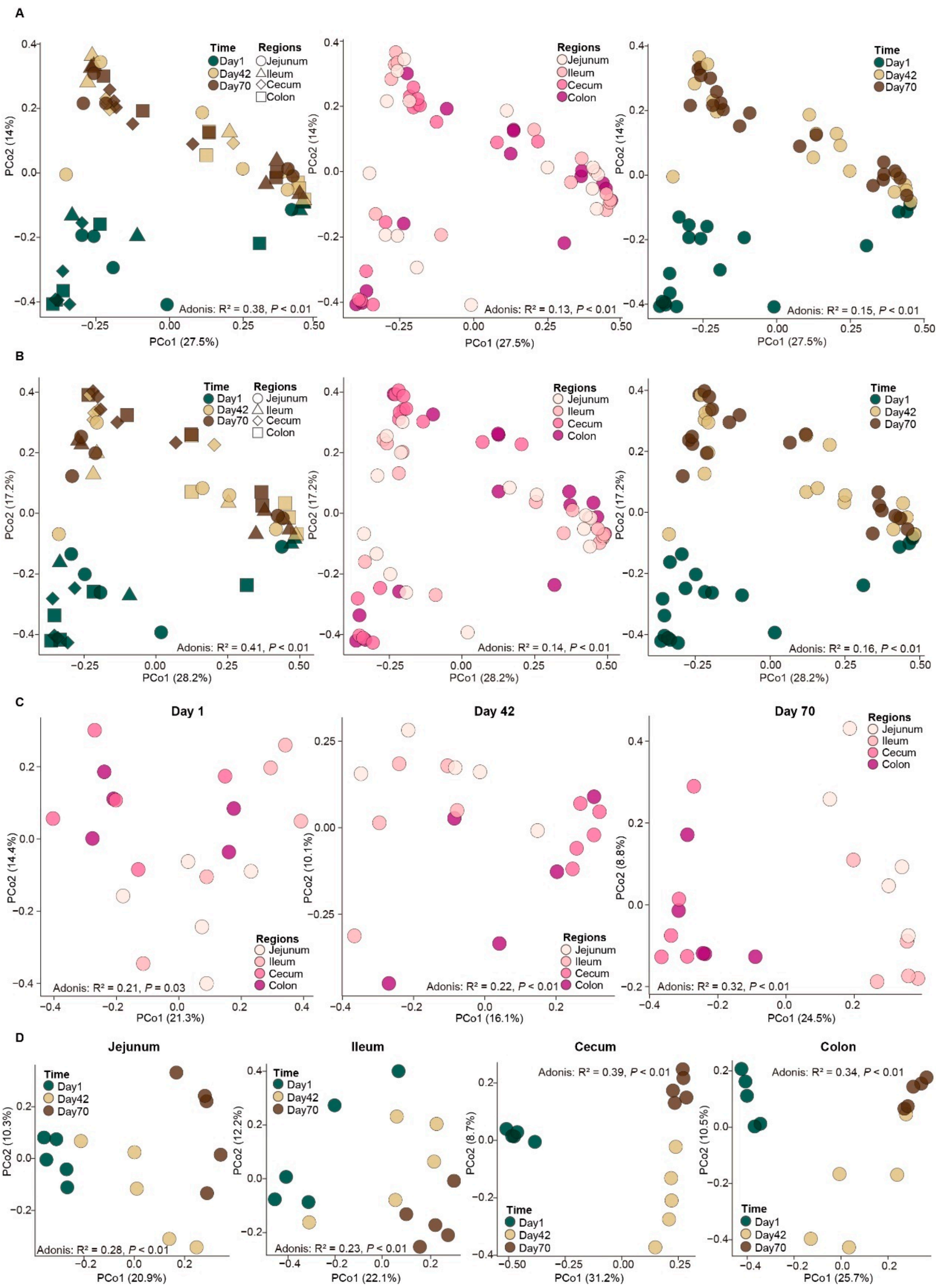


**Figure S1**. PCoA results revealing the changes of the intestinal mucosa microbiota from birth to rumination, and among the different intestinal regions based on Bray-Curtis dissimilarity matrix (**A**) and weighted Unifrac distance (**B**). PCoA results revealing the changes of intestinal mucosa microbiota at the day 1, day 42 and day 70 for the jejunum, ileum, cecum and colon mucosa, respectively,

based on unweighted Unifrac distance. (**C**). PCoA results revealing the changes of intestinal mucosa microbiota in the jejunum, ileum, cecum and colon mucosa at the day 1, day 42 and day 70, respectively, based on unweighted Unifrac distance (**D**).
